# milR4 and milR16 Mediated Fruiting Body Development in the Medicinal Fungus *Cordyceps militaris*

**DOI:** 10.3389/fmicb.2019.00083

**Published:** 2019-01-28

**Authors:** Ying Shao, Jin Tang, Shanglong Chen, Yonghua Wu, Kun Wang, Bin Ma, Qiumei Zhou, Anhui Chen, Yulong Wang

**Affiliations:** ^1^Jiangsu Key Laboratory of Food Resource Development and Quality Safe, Xuzhou University of Technology, Xuzhou, China; ^2^Jiangsu Xuzhou Technician Institute, Xuzhou, China; ^3^Jiangsu KONEN Biological Engineering Co., Ltd., Nanjing, China; ^4^Experimental Center of Clinical Research, The First Affiliated Hospital of Anhui University of Chinese Medicine, Hefei, China; ^5^Anhui Provincial Key Laboratory of Microbial Pest Control, Anhui Agricultural University, Hefei, China; ^6^Key Laboratory of Crop Quality Improvement of Anhui Province/Crop Research Institute, Anhui Academy of Agricultural Sciences, Hefei, China

**Keywords:** microRNA-like RNAs (milRNAs), sexual development, *Cordyceps militaris*, disruption, over-expression

## Abstract

*Cordyceps militaris* readily performs sexual reproduction, thus providing a remarkably rich model for understanding the processes involved in sexual development. It could regulate expression of human genes by diet-derived miRNA-like RNAs (milRNAs). However, the study of miRNAs in *C. militaris* has been limited. In the present study, genes encoding Dicers, Argonautes, and RNA-dependent RNA polymerases were identified. Illumina deep sequencing was performed to characterize the milRNAs in *C. militaris* at asexual and sexual development stages. Total 38 milRNAs were identified and five milRNAs were validated by northern blot and qRT-PCR, out of which, 19 were specific for sexual development. Importantly, the fungi could not form fruiting bodies after disruption of milR4, while the perithecium was formed in advance after over-expression of milR4. Abnormal pale yellow fruiting body primordium, covered with abnormal primordium, was formed in the strain with miR16 disruption. Although no milR4 or milR16 target genes were identified, differential expression of many different genes involved in mycelium growth and sexual development (mating process, mating signaling, and fruiting body development) among these mutants were found. Overall, milRNAs play vital roles in sexual development in *C. militaris*.

## Introduction

MicroRNAs (miRNAs) are small regulatory RNA molecules (18–24 nt) that play a pervasive role in gene regulation and influence a variety of biological processes in animals, plants, viruses, and fungi (Reis, 2017; Zeng et al., 2018). To date, following the discovery of key components of miRNA maturation and function like RNA-dependent RNA polymerase (RDRP), argonaute (AGO), and Dicer proteins in the universal fungal species, hundreds of milRNAs have been identified from dozens of fungal species (Lee et al., 2010; Lau et al., 2013; Meng et al., 2017; Zeng et al., 2018). Fungal miRNA-like RNAs were first discovered in *Neurospora crassa* and produced by at least four different mechanisms that use a distinct combination of factors, including Dicers, QDE-2, the exonuclease QIP, and an RNase III domain-containing protein, MRPL3 (Lee et al., 2010). Subsequently, milRNAs in various species of fungi have been discovered, such as *Sclerotinia sclerotiorum*, *Metarhizium anisopliae*, *Trichoderma reesei*, *Penicillium marneffei*, *Aspergillus flavus*, *Ophiocordyceps sinensis*, *Coprinopsis cinerea*, etc. (Zhou J. et al., 2012; Zhou Q. et al., 2012; Kang et al., 2013; Lau et al., 2013, 2018; Bai et al., 2015; Zhang et al., 2018). Although milRNAs have been found in various fungi, there is limited information on their function and target recognition.

*Cordyceps militaris*, a traditional medicinal mushroom, has been used as a natural invigorant for thousands of years in China (Shrestha et al., 2012). Many components in *C. militaris* have been identified to have therapeutic effects on various diseases; therefore, some purified extracts from *C. militaris* have been recently used for the treatment of several diseases (Yang et al., 2015). A recent report showed that *C. militaris* containing high levels of two milRNAs (milR1321 and milR3188) that target 3’-untranslated region of CXCR2 mRNA to inhibit its expression, alleviates severity of murine acute lung injury, suggesting the regulation of human gene expression by diet-derived milRNAs in *C. militaris*, as reported previously (Liu et al., 2015). However, only two milRNAs in *C. militaris* have been identified thus far, which inhibits the research for potential roles of diet-derived milRNAs on human gene expression.

Sexuality in fungi has long been recognized as one of the most perplexing yet intriguing facets of the biology of this large and varied group of microorganisms. To date, insights have been gained into many aspects of fungal sexuality, following the application of modern molecular genetic techniques. The link between reactive oxygen species (ROS) generation and induction of fruiting bodies in filamentous fungi was established in *A. nidulans*, *Botrytis cinerea*, *S. sclerotiorum*, and *N. crassa* (Lara-Ortiz et al., 2003; Cano-Dominguez et al., 2008; Segmuller et al., 2008; Kim et al., 2011). MAPK signaling modules were identified and they showed important functions on the sexual development of *A. nidulans* and *Podospora anserina* (Wei et al., 2003; Lalucque et al., 2012). Recently, some studies showed that small RNAs have vital roles in fungal sexual development. The sex-specific induced exonic small interference RNA-mediated RNA interference mechanism played an important role in fine-tuning the transcriptome during ascospore formation in the head blight fungus *Fusarium graminearum* (Moran et al., 2017). Sexual development was regulated by the biogenesis of perithecium-specific milRNAs in *F. graminearum* (Zeng et al., 2018). Although it is clear that milRNAs are indispensable in fungal sexual development, no study has reported that which genes were regulated by milRNAs in the process of sexual reproduction in fungi.

In this study, Dicer, AGO and RDRP genes of *C. militaris* were identified and their expression profiles at different developmental stages were detected. To gain insight into the regulatory role of milRNAs in the sexual development of fungi, we predicted the sexual and asexual development-related milRNAs via deep sequencing and *in silico* analysis. Two milRNAs in *C. militaris* were examined in detail, to investigate their functions in sexual development.

## Materials and Methods

### Fungal Strains

The fungal strain used in this study was *C. militaris* PM53 (Chinese General Microbiological Culture Collection no. 3.15517), which contains both *MAT1-1* and *MAT1-2* mating types. The mycelia of this strain were inoculated into a 250-ml flask containing 50 ml of liquid PD medium (20% potato and 2% dextrose, w/v). The flask was then incubated at 23°C in a 150-rpm shaker for 7 days. Chinese Tussah silk moth pupae were inoculated with fungal culture in the same flask, cultivated in the dark at 23°C for 5 days, and then kept at 23°C under a 17:7 h dark/light cycle. Single ascospore isolates were randomly isolated from the discharging fruiting bodies to collect opposite MAT isolates as previously described (Chen et al., 2017). Strains PM53-1 (*MAT1-1*) and PM53-2 (*MAT1-2*) were selected for further study.

### Media and Growth Conditions

Conidia were harvested in a 0.05% Tween-80 aqueous solution. This conidial suspension was then filtered through sterile non-woven fabric to remove mycelia, which were then washed with sterile water. The final spore concentration (10^5^ spores mL^-1^) was determined by direct counting using a hemocytometer. For the collection of different samples in asexual development, mixed conidia of PM53-1 and PM53-2 at ratio of 1:1 was inoculated onto PDA plates (20% potato, 2% dextrose and 1.5% agar, w/v) and incubated at 23°C under a 12 h:12 h light/dark cycle. For fruiting body production, mixed conidia of PM53-1 and PM53-2 at ratios of 1:1 were inoculated into a 250-ml flask as mentioned above.

### Identification and Real-Time PCR Analysis of RDRP, Dicer and AGO Families

Genes encoding RDRP, Dicer, and AGO proteins in the fungal genome^1^ were identified through BLAST search (Expect threshold = 10). Sequences of RDRP-1/2/3, Dicer-1/2 and AGO-1/2/3 of *N. crassa* as reference sequences to retrieve their orthologs in fungi (Hu et al., 2013). The amino acid sequence of RDRP, Dicer, and AGO proteins from *C. militaris* and other fungi obtained from GenBank were aligned using CLUSTALW (Thompson et al., 1994). Phylogenetic tree analysis was performed using the Maximum Likelihood Method based on the Tamura-Nei model and 1000 bootstrap replicates with MEGA 7.0 software (Kumar et al., 2016).

Total RNA was extracted at different asexual developmental stages of *C. militaris*, i.e., 3-day-old (A3), 9-day-old (A9), and 15-day-old (A15), as well as from different sexual developmental stages, i.e., nascent (SNF), stalk formation (SMIF) and mature fruiting bodies (SMAF) as reported previously (Wang et al., 2015). First-strand cDNA was synthesized with a PrimeScript^TM^ II 1st Strand cDNA Synthesis Kit (Takara) according to the manufacturer’s instructions. The resulting cDNA templates were used for quantitative RT-PCR amplification with a SYBR Green kit (Takara, Dalian, China) and Bio-Rad CFX96 system (Bio-Rad, CA, United States). All sampling were performed in triplicates. RT-PCR data were analyzed using the 2^-ΔΔCt^ method of relative quantification, using glyceraldehyde-3-phosphate dehydrogenase (GAPDH) as the internal control for each sample. Six independent experiments were performed and all data are presented as the mean ± SE of six replicates. Details of primers used in this study are given in Supplementary Table S1.

### RNA Isolation and Library Construction

A9 and SMAF were selected to construct small RNA and cDNA libraries of asexual and sexual stage, respectively. Total RNA was extracted using the RNAiso Plus reagent (Takara, Shiga, Japan) according to the manufacturer’s instructions and then treated with RNase-free DNase I. RNA concentration was then evaluated by a NanoDrop ND-2000 spectrophotometer (NanoDrop Technologies, Wilmington, DE, United States) and Agilent 2100 Bioanalyzer (Agilent, United States). Small RNAs (15–30 nt) were extracted from total RNA on a 15% denaturing polyacrylamide gel and ligated to specific 5′ adaptor and 3′ adaptor samples. After reverse transcription, the cDNA libraries were sequenced (PE100) on an Illumina HiSeq 2000 platform (BGI, Shenzhen, China). For each strain, three biological replicates were used and raw data were deposited in the NCBI Sequence Read Archive database with accession code PRJNA496418.

### Data Analysis of Small RNA

High-quality small RNA reads were obtained from raw reads by filtering out poor-quality reads and removing adaptor sequences using the FASTX toolkit using default settings (Blankenberg et al., 2010). Adaptor-trimmed unique sequences were aligned to the *C. militaris* genome using bowtie (Langmead et al., 2009; Zheng et al., 2011). After removal of known non-coding RNAs (rRNA, tRNA, snRNA, and snoRNA) by BLAST or Bowtie, the unannotated small RNAs were used for novel miRNA prediction by using mireap (Li et al., 2012). The raw abundance of milRNAs was normalized according to transcripts per million (TPM) normalization (t Hoen et al., 2008). PsRobot and TargetFinder were used for the prediction of miRNAs and their targets according to plant-like target interactions, and based on the methods described previously (Zeng et al., 2018).

### Expression Analysis for milRNAs, Targets, Asexual and Sexual Development-Related Genes

The stem-loop real-time PCR method was used to quantitate milRNA expression in this study using 5S rRNA as the internal control for each sample (Zhou Q. et al., 2012). Northern blot analysis of milRNA identification was performed according to the methods described previously (Lau et al., 2018). Asexual and sexual development-related genes were selected from previous study, the expression of targets and their genes was detected as mentioned above, and GAPDH was used as the internal control for each sample (Zheng et al., 2011). Six independent experiments were performed and all data were presented as the mean ± SE of six replicates. Details of primers used in this study are given in Supplementary Table S1. Details of asexual/sexual development-related genes used for qRT-PCR were listed in Supplementary Table S1.

### Disruption and Over-Expression of milR4 and milR16

Since the mature milR4 is located in the coding region of CCM_00776, in order to delete milR4, its location sequences (5′-A GTC CGA CGA CGA GGA GCC-3′) were changed (5′-A ATC ACT ACT ACT ACT TCC-3′) based on the degeneracy of codons. In brief, milR4 upstream, milR4 downstream, and CCM_00776 3′ flanking regions were amplified, using WT genomic DNA as a template. The trpC sequence from *A. nidulans* was used as the terminator for CCM_00776 transcription. All PCR fragments were individually inserted into pDHt-SK-bar with ClonExpress MultiS One Step Cloning Kit (Vazyme, Nanjing, China). For milR16 deletion, the 5′ and 3′ flanking regions of Pre-milR16 were amplified using WT genomic DNA as a template, and then individually inserted into pDHt-SK-bar with ClonExpress MultiS One Step Cloning Kit (Vazyme, Nanjing, China).

For over-expression of milR4 and milR16, precursor miRNAs of milR4 and milR16 were amplified using WT genomic DNA as a template and individually inserted into pDHt-SK-bar-gpda (containing *gpdA* promoter with *trpC* terminator from *A. nidulans*) with ClonExpress MultiS One Step Cloning Kit (Vazyme, Nanjing, China).

All mutants (PM53-1/2 of Knock-milR4, Knock-milR16, Over-milR4, and Over-milR16) were constructed by means of *Agrobacterium tumefaciens*-mediated transformation (ATMT) as previously described (Wang et al., 2017). All mutants were confirmed by PCR and sequencing. Details of primers used in this study are given in Supplementary Table S1.

## Results

### Phylogenetic and Expression Profiles Analyses of AGO, Dicer and RDRP Genes

**FIGURE 1:**
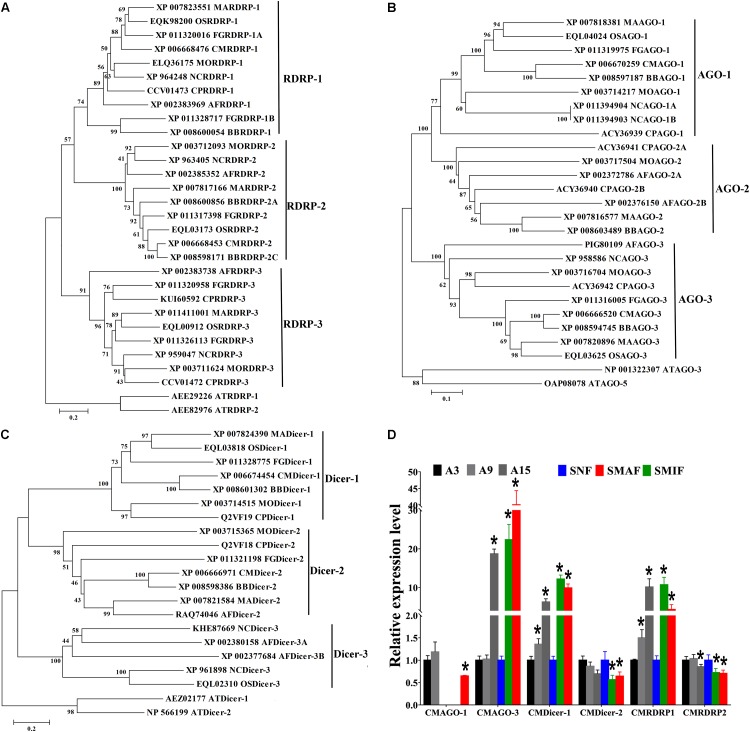
Phylogenetic analysis and expression profiles of Dicers, RDRPs, and AGOs in *C. militaris* and other fungi. Phylogenetic trees constructed with fungal Dicer **(A)**, RDRP **(B)**, and AGO **(C)** proteins. CM, *Cordyceps militaris*; BB, *Beauveria bassiana*; MA, *Metarhizium robertsii*; OS, *Ophiocordyceps sinensis*; MO, *Magnaporthe oryzae*; FG, *Fusarium graminearum*; CP, *Cryphonectria parasitica*; NC, *Neurospora crassa*; AF, *Aspergillus flavus*; AT, *Arabidopsis thaliana*. Expression profiles of Dicers, RDRPs, and AGOs at different developmental stages of *C. militaris* **(D)**. Total RNAs were extracted at different asexual developmental stages of *C. militaris*, i.e., 3-day-old (A3), 9-day-old (A9), and 15-day-old (A15), and from different sexual developmental stages, i.e., nascent (SNF), stalk formation (SMIF), and mature fruiting bodies (SMAF). Data are expressed as means ± SE of the values from six independent experiments. Student’s *t*-test was used to determine the statistical significance of differences between groups. The asterisk “^∗^” represents a significant difference level of *P* < 0.05 compared with A3 (asexual development) or SNF (sexual development).

RNA-dependent RNA polymerase, AGO, and Dicer proteins are key components of miRNA maturation and function in eukaryotes. Possessing of these proteins could be an evidence for the presence of miRNAs in *C. militaris*. Therefore, RDRP, AGO, and Dicer genes in *C. militaris* were identified. Genome of *C. militaris*^2^ was searched and two RDRPs, two AGOs, and two Dicers were identified. To annotate of these genes, Phylogeny trees were generated with RDRP, AGO, and Dicer genes from other eight fungal species, including the entomopathogenic fungi (*M. anisopliae*, *Beauveria bassiana*, *O. sinensis*), plant pathogenic fungi (*Magnaporthe oryzae*, *F. graminearum*, *Cryphonectria parasitica*), the model filamentous fungus *N. crassa*, a saprophytic filamentous fungus *A. flavus*. Phylogenetic analysis showed the number of AGO genes varied from two (*C. militaris*, *O. sinensis*) to four (*Cryphonectria parasitica*), but most of the fungal species examined here possessed three AGO genes. Fungal species examined here possessed two Dicer genes, except *A. flavus* possessed four Dicer genes. The number of RDRP genes varied from two (*C. militaris*) to five (*F. graminearum*), but most of the fungal species examined here possessed three RDRP genes. Therefore, two RDRPs (CMRDRP1 and CMRDRP2), two AGOs (CMAGO-1 and CMAGO-3), and two Dicers (CMDicer-1 and CMDicer-2) were designated based on phylogenetic analysis in *C. militaris* (Figure 1A–C).

To further explore the possible roles of milRNAs at different developmental stages, RDRP, AGO, and Dicer gene expression profiles at different developmental stages were studied by quantitative real-time PCR. The results showed that the expression levels of all genes except CMAGO-1 gene were detected at all investigated developmental stages (Figure 1D). In particular, the expression trends of CMAGO-3, CMDicer-1, and CMRDRP1 were similar at each stage. The highest gene expression levels of asexual development were seen in A15, whereas, in sexual development, the gene expression levels were significantly higher in SMIF and SMAF than in SNF. The transcript levels of these genes expressed significant different throughout the developmental stages, suggesting milRNA expression and functions were variations during development. Therefore, A9 (as a control) and SMAF of *C. militaris* were chosen for sRNA sequencing, because the expression levels of RDRP, AGO, and Dicer genes were detected in A9 and SMAF stages.

### milRNAs in Asexual and Sexual Development Stages

To identify the milRNAs associated with sexual development, the transcriptome of small RNAs in asexual (A9, as a control) and sexual (SMAF) developments were sequenced in triplicate, which were named as AS-1, AS-2, AS-3 (A9, asexual development) and S-1, S-2, S-3 (SMAF, sexual development). Approximately 2 million clean reads were obtained at each sample after quality filtering for low quality reads and trimming for adaptor sequences (Supplementary Table S2). A total of thirty-eight milRNAs were identified at different developmental stages of *C. militaris* (Supplementary Table S3). Sequence search of *C. militaris* milRNAs in miRBase and other milRNAs reported in other fungi found no homologous sequence in any other organism including filamentous fungi, indicating a species-specific feature of *C. militaris* milRNAs. To validate the sequencing data, the expression levels of thirty-eight milRNAs were determined using stem-loop qRT-PCR, five milRNAs were chosen and validated by northern blot (Supplementary Figure S1).

### Comparison Between milRNAs in *C. militaris* With Other Fungi

**FIGURE 2:**
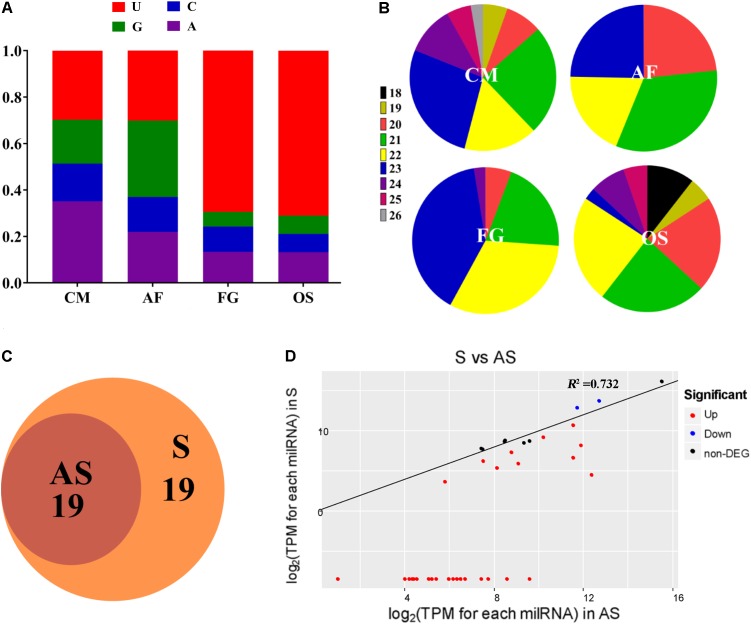
Characteristics and expression of milRNAs in *C. militaris*. **(A)** 5′-terminal nucleotide of milRNAs among different fungi. CM, *Cordyceps militaris*; OS, *Ophiocordyceps sinensis*; AF, *Aspergillus flavus*; FG, *Fusarium graminearum*. The *y*-axis shows the proportion of 5′-terminal nucleotides consisting of G/C/A/U. **(B)** The percentages of size distributions of the milRNAs among different fungi. **(C)** The Venn diagram illustrates the number of milRNAs from asexual or sexual development stages. AS, asexual development; S, sexual development. **(D)** The expression levels of milRNAs in sexual and asexual development stages.

To explore the characteristics of milRNAs in *C. militaris*, milRNAs reported in other fungi (the entomopathogenic fungus *O. sinensis*, the plant pathogenic fungus *F. graminearum*, the saprophytic filamentous fungus *A. flavus*) were re-identified based on the methods mentioned above, and then compared with milRNAs in *C. militaris* (Bai et al., 2015; Wang et al., 2016; Zhang et al., 2018). Total 73, 157, and 38 milRNAs were obtained from *A. flavus*, *F. graminearum*, and *O. sinensis*, respectively (Supplementary Table S3). The results from nucleotide bias analysis of milRNAs from different fungi showed that the first base at the 5′ end of milRNA had a strong preference for “A” in *C. militaris*, which is different for *A. flavus* (G), *F. graminearum*, and *O. sinensis* (U) (Figure 2A). Analysis of the length distribution of these milRNAs revealed that they ranged from 18–30 nt. In *C. militaris* and *F. graminearum*, the number of 21–23 nt long milRNAs was higher than the number of milRNAs with other lengths, while in *A. flavus* and *O. sinensis* the numbers of 20–23 nt and 20–22 nt long milRNAs, respectively, were higher than the number of milRNAs with other lengths (Figure 2B).

### Differential Expression of milRNAs in *C. militaris*

The abundance of milRNAs was normalized according to transcripts per million (TPM) normalization, where the n-base is 1,000,000. Among 38 milRNAs, 19 milRNAs were expressed exclusively in the sexual developmental stage, while the other 19 milRNAs were expressed in both asexual as well as sexual developmental stages, suggesting that the sexual development-specific milRNAs play a crucial role during sexual development (Figure 2C). In the present study, we used an absolute value of the log_2_ ratio ≥ 1 (*p* < 0.05) as the threshold to determine the significance of differences in milRNA expression, and a total of 31 milRNAs were found to have significantly different expression (Figure 2D and Table 1). Among them, only two milRNAs (milR16 and milR34) were down-regulated, while other milRNAs (rest of the 29 milRNAs) were up-regulated in the sexual developmental stage, when compared with their expression in asexual developmental stage.

**Table 1:** Differentially expressed milRNAs at sexual (S) and asexual development (AS) stages.

miRNA ID	Expression in AS	Expression in S	log_2_Ratio (S/AS)	*P*-value
milR2	4.26	18.54	2.82	8.99E-88
milR3	97.58	1251.15	4.39	0
milR4	0.001^∗^	253.82	13.59	0
milR5	119.09	215.71	1.47	0
milR6	0.001^∗^	30.39	10.61	2.08E-163
milR7	52.66	145.7	2.01	0
milR9	0.001^∗^	7.02	8.46	1.40E-50
milR10	0.001^∗^	57.07	11.42	6.09E-249
milR11	0.001^∗^	6.12	8.26	3.08E-45
milR12	0.001^∗^	7.75	8.47	5.64E-51
milR13	0.001^∗^	127.42	12.63	0
milR14	13.73	92.34	3.29	0
milR16	21881.14	3608.64	-1.49	0
milR17	0.001^∗^	12.29	9.15	4.20E-74
milR18	0.001^∗^	6.12	8.02	1.97E-39
milR19	139.1	254.17	1.57	0
milR20	0.001^∗^	26.94	10.38	1.66E-144
milR21	0.001^∗^	34.19	10.72	1.45E-172
milR22	25.91	60.98	2.3	6.38E-226
milR23	0.001^∗^	5.33	8.06	2.05E-40
milR24	20.17	181.4	3.82	0
milR25	0.001^∗^	0.67	5.17	2.23E-07
milR26	0.001^∗^	6.74	8.3	5.41E-47
milR27	0.001^∗^	11.22	8.92	2.24E-65
milR28	0.001^∗^	20.86	9.92	6.43E-113
milR29	0.001^∗^	14.01	9.43	1.10E-86
milR30	191.82	393.19	1.87	0
milR31	0.001^∗^	23.75	10.22	8.61E-133
milR32	7.74	1741.13	9.34	0
milR34	148.98	22.17	-1.66	2.04E-178
milR35	0.001^∗^	70.78	11.82	2.25E-306


*^∗^Indicates no milRNA expression.*

### Disruption and Over-Expression of milR4 and milR16

**FIGURE 3:**
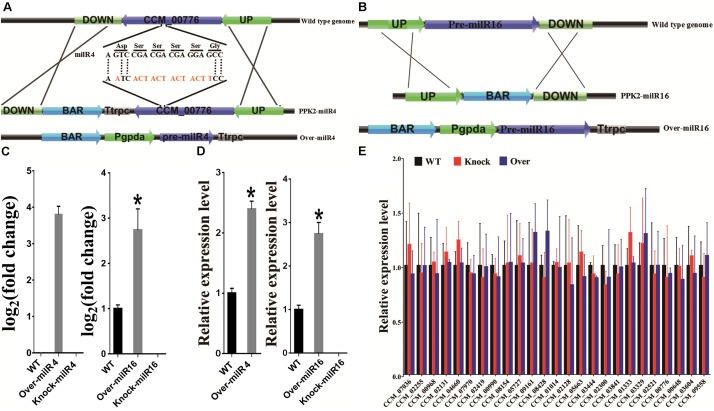
Construction of milR4 or milR16 disruption and over-expression mutants. **(A)** Schematic representation of the plasmids that were used for disruption and over-expression of the milR4. milR4 location sequences of (5′-A GTC CGA CGA CGA GGA GCC-3′) were changed to (5′-A ATC ACT ACT ACT ACT TCC-3′) based on the degeneracy of codons. **(B)** Schematic representation of the plasmids that were used for disruption and over-expression of the milR16. **(C)** Real-time PCR analysis of milR4 and milR16 expression from mycelium wild type strain and different mutants. Mixed conidia of PM53-1 (*MAT1-1*) and PM53-2 (*MAT1-2*) at ratios of 1:1 was inoculated onto PDA plates and incubated at 23°C under a 12 h:12 h light/dark cycle for 14 days for RNA extraction. **(D)** Real-time PCR analysis of milR4 and milR16 expression from fruiting bodies (52 days) of wild type strain and different mutants. N, no fruiting body formation for the detection. **(E)** Analysis of the relative expression levels of milR4 and milR16 predictive miRNA target genes in wild type strain and different mutants. Data are expressed as means ± SE of values from six independent experiments. The asterisk “^∗^” represents a significant difference level of *P* < 0.05.

To explore whether milRNAs play any role in sexual development, milR4 and milR16 in PM53-1 and PM53-2, respectively, were knocked-out and over-expressed (Figure 3A,B and Supplementary Figure S2). Expression levels of milR4 and milR16 were detected by incubating WT and mutant cultures of PM53-1 and PM53-2 at ratios of 1:1 inoculated onto PDA plates. Results showed that no expression of milR4 RNA in mycelium from the wild type (WT) and knock out (Knock-milR4) strains, however, it was expressed in the over-expressed strain (Over-milR4) (Figure 3C). No expression of milR16 RNA in knock out strains (Knock-milR16), while its expression detected in the over-expressed strain (Over-milR16) was higher than that in the WT. The expression levels of milR4 and milR16 were also analyzed in the fruiting bodies of different strains except Knock-miR-4, and both milR4 and milR16 were expressed more than 2.5-folds in the over-expression strains than that in WT, while no milR16 was detected in Knock-miR-16 (Figure 3D).

Since milR4 is located in the coding region of CCM_00776, sequences of milR4 were changed based on the degeneracy of codons, and the expression of CCM_00776 in Knock-milR4 and the WT was detected by real-time PCR for ensuring that expression of CCM_00776 was not affected by the sequence change (Supplementary Figure S3). No significant expression difference (*P* = 0.3724) was detected between the wild type strain and Knock-milR4, suggesting the expression of CCM_00776 was not affected after the milR4 sequence change.

### milR4 and milR16 Are Indispensable for the Fungal Sexual Development

**FIGURE 4:**
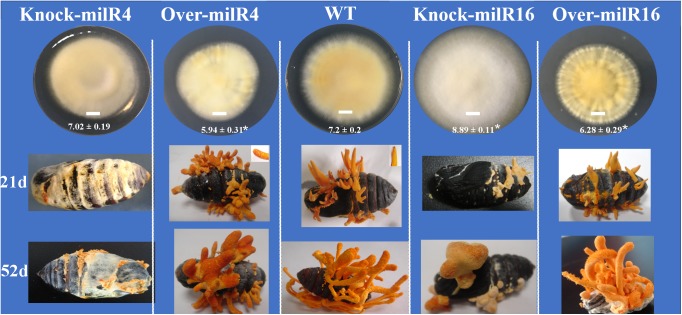
Asexual and sexual development of the wild type and different mutant strains. Bar = 1 cm. The diameters of different strains are showed under the Bar. The asterisk “^∗^” represents a significant difference level of *P* < 0.05 compared to WT.

The effect of disrupting and over-expressing different milRNAs on the vegetative growth was examined by incubating WT and mutant cultures of PM53-1 and PM53-2 at ratios of 1:1 inoculated onto PDA plates. As compared to the WT strain, the growth rate of the Over-milR4 and Over-milR16 were markedly reduced by 17.5% (*p* < 0.05) and 12.8% (*p* < 0.05), respectively, whereas the growth rate of Knock-miR-16 was increased by 23.4% (*p* < 0.05) (Figure 4). Knock-miR-16 aerial mycelia were white colored while Over-milR16 aerial mycelia were yolk-yellow and much deeper in color than that of the WT strain.

The WT and mutant liquid cultures of PM53-1 and PM53-2 at ratios of 1:1 were injected into silk moth pupae to determine whether the milRNAs regulate the development of fruiting bodies. Among all the tested strains, only WT and Over-milR16 strains produced normal nascent (21 days) and mature (52 days) fruiting bodies (Figure 4). In contrast, yellow mycelia formed and covered the pupae inoculated with the Knock-miR-4 at 21 days, and normal primordia did not form even after a long period. When inoculated with the Over-milR4, the primordia were formed; however, many perithecia were formed at 21 days, clearly inhibiting the growth of the fruiting body. When inoculated with the Knock-miR-16, abnormal primordia with pale yellow color were formed at 21 days and fruiting bodies were blocked and covered with abnormal perithecia.

### Transcriptional Expressions of Different milRNA Target Genes

Potential target genes of milR4 and milR16 were predicted to better understand their probable roles in sexual development (Supplementary Table S4). The sexual development-specific milR4 was predicted to target 24 genes including transcripts encode fungal transcriptional regulatory protein (CCM_08428), frequency clock protein (CCM_01014), and WD repeat-containing protein (CCM_02300). Whereas, milR16 was predicted to target a transcript encode putative glycosyltransferase (CCM_ 09558).

miRNAs directly degrade their target mRNA in plants and mediate suppressed mRNA translation in plants and animals (Huntzinger and Izaurralde, 2011; Reis, 2017). Indeed, expression levels of 24 target genes of milR4 and 1 target gene of milR16 were analyzed in 9-day-old mycelia of different mutants. No significant difference in the expressed genes was detected between the mutants (Knock and Over) and the WT, suggesting that these target gene expressions were not regulated by these milRNAs, and these predicted target genes were not “really” target genes of milR4 and milR16 (Figure 3E).

### Expressions of Asexual and Sexual Development-Associated Genes in Different Strains

To further explore the mechanism of milRNA function on the asexual and sexual development of *C. militaris*, some genes associated with asexual or sexual development were chosen based on a previous report (Zheng et al., 2011).

**FIGURE 5:**
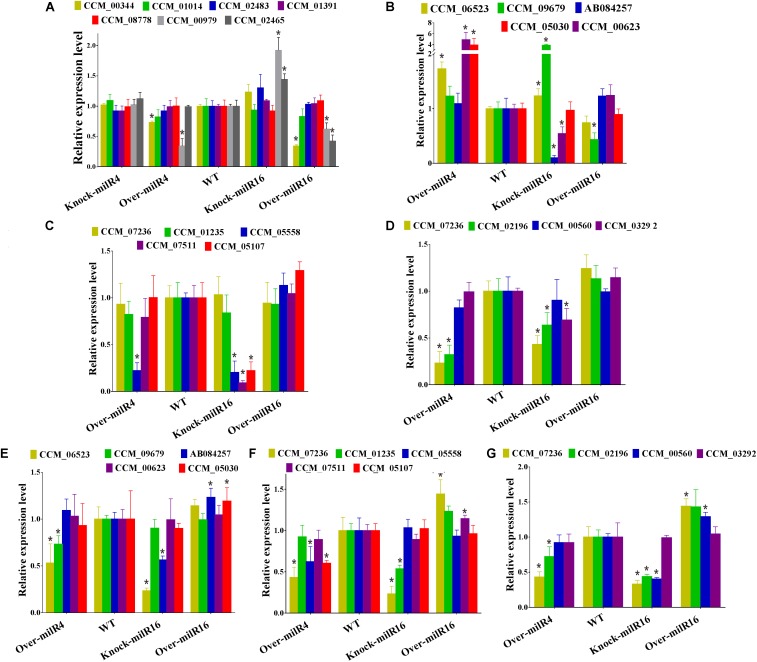
Analysis of the relative expression levels of genes involved in asexual or sexual development of different strains. **(A)** Expression levels of seven asexual development-associated genes. Expression levels of five genes for mating process **(B)**, five genes for mating signaling **(C)** and four genes for fruiting body development **(D)** from 21 days old cultured strains. Expression levels of five genes for mating process **(E)**, five genes for mating signaling **(F)**, and four genes for fruiting body development **(G)** from 52 days old cultures strains. All data are expressed as means ± SE of the values from six independent experiments. The asterisk “^∗^” represents a significant difference level of *P* < 0.05.

Expression levels of seven asexual development-associated genes were studied in different strains (Figure 5A). The results showed that two genes (CCM_00344 and CCM_00979) in Over-milR4 and three genes (CCM_00344, CCM_00979, and CCM_02465) in Over-milR16 showed significantly lower expression, while two genes (CCM_00979 and CCM_02465) in Knock-miR-16 showed significantly higher expression compared to that in the WT. However, all the seven genes were expressed at similar levels in the WT. These data agree with the data showing that Over-milR4 and Over-milR16 grow slower while Knock-miR-16 grows faster than the WT.

Five mating process, five mating signaling, and four fruiting body development related genes were used to detect their expression levels in 21- and 52-day-old cultures of Over-milR4, Knock-miR-16, and Over-milR16 strains (Figure 5B–G). At 21 days, out of five mating process genes, 3 genes were up-regulated in Over-milR4, 2 genes were up-regulated and 2 genes were down-regulated in Knock-miR-16, and only 1 gene was down-regulated in Over-milR16 (Figure 5B). Out of five mating signaling genes, 1 and 3 genes were down-regulated in Over-milR4 and Knock-miR-16, respectively (Figure 5C), whereas, out of four fruiting body development genes, 2 and 3 genes were down-regulated in Over-milR4 and Knock-miR-16, respectively (Figure 5D). At 52 days, out of five mating process genes, 2 and 2 genes were down-regulated in Over-milR4 and Knock-miR-16, respectively, while 2 genes were up-regulated in Over-milR16 (Figure 5E). Out of five mating signaling genes, 3 and 2 genes were down-regulated in Over-milR4 and Knock-miR-16, respectively, while 2 genes were up-regulated in Over-milR16 (Figure 5F). Whereas, out of four fruiting body development genes, 2 and 3 genes were down-regulated in Over-milR4 and Knock-miR-16, respectively, while 2 genes were up-regulated in Over-milR16 (Figure 5G). These data indicated that genes associated with sexual development were regulated indirectly by milRNAs.

## Discussion

milRNAs in various species of fungi have been discovered thus far; however, there is little information on their functions. An important traditional medicinal mushroom, *C. militaris*, was used to explore the functions of milRNAs in the sexual development of fungi. In *N. crassa*, milRNAs are produced by at least four different mechanisms that use a distinct combination of factors, Dicers and RDRPs, which were closely related to those of in *C. militaris*, indicating multiple milRNA biogenesis mechanisms existed in *C. militaris* (Lee et al., 2010). Many genes were expressed at different developmental stages or in specific organs, or both, which provides information regarding their functions. In the present study, we analyzed the expression of CMDicers, CMAGOs and CMRDRPs by relative qRT-PCR at different developmental stages of *C. militaris*. CMDicer-1, CMRDRP1, and CMAGO-3 were expressed with significant differences among different development stages, suggesting that CMDicer-1 and CMRDRP1 are the major genes that process dsRNA into mature milRNA, and CMAGO-3 is the major gene involved in the milRNA functioning in asexual and sexual development. Interestingly, CMAGO-1 did not express at A15, SNF, and SMIF, indicating CMAGO-1 is not necessary for the fungal development at these developmental stages.

Thirty-eight novel milRNAs were discovered by high-throughput sequencing, 19 milRNAs were expressed exclusively in the sexual developmental stage while the other 19 milRNAs were expressed in both asexual as well as sexual developmental stages. In *F. graminearum*, 49 milRNAs were detected in the asexual stage, and 143 milRNAs were detected in the sexual stage, which is consistent with *C. militaris* that more milRNAs were needed for sexual development than for asexual development in fungi (Chen et al., 2015; Zeng et al., 2018).

To explore the roles of milRNA in sexual development of the fungi, milR4 and milR16 were knocked out and over-expressed in this study. In animals and plants, miRNAs negatively regulated gene targeting by seed-region matching and near-perfect matching (Huntzinger and Izaurralde, 2011; Ueda et al., 2018). Several software (miRanda, PITA, microTar, PsRobot, and TargetFinder et al.) were tried for milRNA target gene prediction, we finally found that expression of these targets predicted by PsRobot and TargetFinder showed very weak correlation (*p* = 0.6045) with expression of these milRNAs (data unpublished), therefore, PsRobot and TargetFinder were used to predict milRNA target genes in this study. However, no significant differences in milR4 and milR16 target gene expression levels were detected between the mutants (Knock and Over) and WT, revealing that the expression of the target genes was not regulated by these milRNAs. Therefore, to study the fungal milRNA target genes, methods to be used should be different from those used in plants and animals, which are our targets for future functional studies.

The growth rates of the Over-milR4 and Over-milR16 were markedly reduced, while those of Knock-miR-16 was increased compared to the WT strain, indicating that over-expressed milR4 or milR16 may inhibit some genes involved in the fungal asexual development. Although no “really” target genes of milRNAs were identified, expression of seven asexual development-associated genes were detected to further explore milRNA functions on the fungal development. Real-time PCR results showed that some genes involved in vegetative growth were negative regulated by milR4 and milR16. These data indicated that milRNAs could control fungal growth by indirect regulation of gene expression.

All the mutants and wild type were injected in pupae to examine milRNA functions in the sexual development of the fungi. Circular (circRNAs) and long non-coding RNAs (lncRNAs) could act as miRNA sponges, consequently repress their regulatory effect in eukaryotes (Kulcheski et al., 2016; Paraskevopoulou and Hatzigeorgiou, 2016). Among the tested strains, only the wild type and Over-milR16 produced normal nascent and mature fruiting bodies, suggesting these circRNA/lncRNA-miRNA mechanisms were activated to prevent the functions of overexpressed milR16. Interestingly, yellow mycelia formed and covered on pupae inoculated with the Knock-miR-4 at 21 days, revealed that the fungi lost the ability to form normal nascent fruiting bodies and that milR4 is indispensable for the fungal sexual development. Many perithecium-specific milRNAs have been found which may have vital roles in ascospore discharge of sexual development in *F. graminearum* (Zeng et al., 2018). In the present study, when inoculated with the Over-milR4, many perithecia were formed in advance and the growth of fruiting bodies was clearly inhibited, indicating that milR4 regulates the production of perithecia in the fungal development. Pigments were important for the sexual development as well as secondary metabolism production in *C. militaris*. When inoculated with the Knock-miR-16, abnormal primordia with pale yellow color formed and fruiting body were blocked and covered with abnormal perithecia. Knock-miR-16 aerial mycelia were white while Over-milR16 mycelia were of much deeper yolk yellow in color compared to that of the wild-type strain, suggesting that some genes involved in pigment production were indirect regulated by milR16 (Yang et al., 2015).

## Conclusion

This study is the first report on genome-wide analysis of milRNAs in asexual/sexual development stages of *C. militaris*. Firstly, putative core miRNA biogenesis and function proteins, Dicer, RDRP, and AGO, in the fungal genome were identified, phylogenetic analysis showed that these proteins were closely related to those in other fungal species. Moreover, 31 milRNAs were found significantly different expressed in different development stages. Importantly, abnormal fungal asexual and sexual development phenotypes were found after disruption/over-expression of milRNAs in *C. militaris*. Altogether, these results suggested that milRNAs play important roles in the regulation of the fungal development, contribute to milRNA function mechanisms among different kingdoms.

## Author Contributions

AC, YlW, and QZ conceived and designed the study. YlW wrote the manuscript. YS and JT conducted the experiments. SC and YhW analyzed the data. KW and BM performed some of the experiments. All authors read and approved the final manuscript.

## Conflict of Interest Statement

KW and BM were employed by Jiangsu KONEN Biological Engineering Co., Ltd. The remaining authors declare that the research was conducted in the absence of any commercial or financial relationships that could be construed as a potential conflict of interest.

## Footnotes

1 https://www.ncbi.nlm.nih.gov/genome/?term=Cordyceps+militaris+CM01

2 https://www.ncbi.nlm.nih.gov/genome/?term=militaris
