# Supplementary material for: milR4 and milR16 Mediated Fruiting Body Development in the Medicinal Fungus Cordyceps militaris
Source: Front Microbiol. 2019 Jan 28;10:83. doi: 10.3389/fmicb.2019.00083 (PMC6362416; doi:10.3389/fmicb.2019.00083)
Supplement: Supplementary file 1 [file Data_Sheet_1.docx]

Supplementary Material

milR4 and milR16 mediated fruiting body development in the medicinal fungus *Cordyceps militaris*

Ying Shao^1†^, Jin Tang^2†^, Shanglong Chen^1^, Yonghua Wu^1^, Kun Wang^3^, Bin Ma^3^, Qiumei Zhou^4^, Anhui Chen^1*^, Yulong Wang^5,6*^

*** Correspondence:** Corresponding Authors: chenah201@163.com (AH Chen); yulongwa@mtu.edu (YL Wang)

# Supplementary Figures and Tables

##
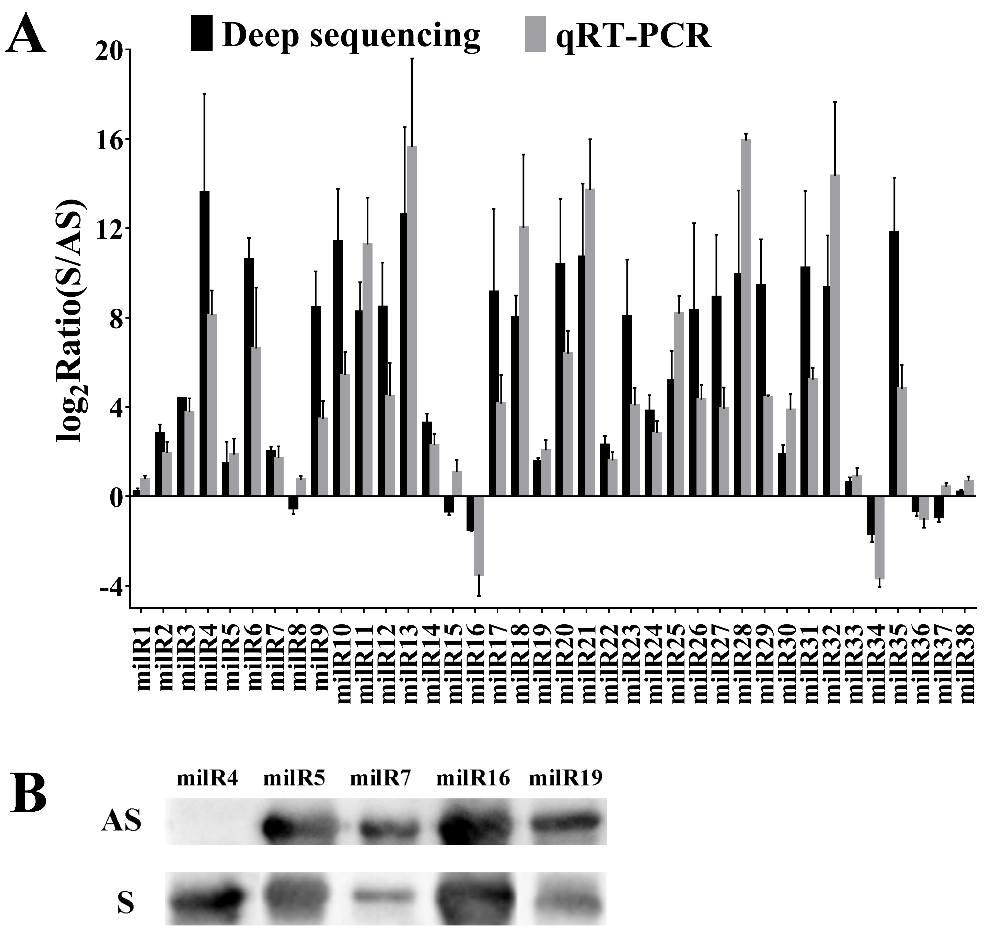
Supplementary Figures

**Supplementary Figure 1.** qRT-PCR and northern blot validation of the putative milRNAs in *C. militaris*. (A) qRT-PCR validation of the 38 putative milRNAs. (B) The 5 putative milRNAs were chosen for northern blot validation in asexual and sexual development stages.


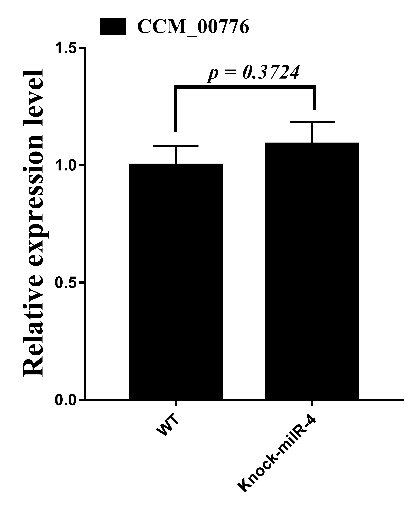

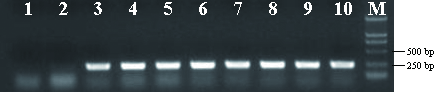
**Supplementary Figure 2.** Confirmation of different mutants by PCR. 256-bp fragments from the BAR coding region were PCR-amplified to confirm these mutants. 1, wild type PM53-1; 2, wild type PM53-2; 3, Knock-milR4 from PM53-1; 4, Knock-milR4 from PM53-2; 5, Over-milR4 from PM53-1; 6, Over-milR4 from PM53-2; 7, Knock-milR16 from PM53-1; 8, Knock-milR16 from PM53-2; 9, Over-milR16 from PM53-1; 10, Over-milR16 from PM53-2.

**Supplementary Figure 3.** Real-time PCR analysis of CCM_00776 in the wild type strain and Knock-milR4.

## Supplementary Tables

**Supplementary Table 1.** PCR primers used in this study.

**Supplementary Table 2.** General features of sRNA sequencing of C. militaris

**Supplementary Table 3.** Prediction of microRNA-like RNAs in different fungi.

**Supplementary Table 4.** Predicted milRNA targets in *C. militaris*.
